# Design of a Novel Oxygen Therapeutic Using Polymeric Hydrogel Microcapsules Mimicking Red Blood Cells

**DOI:** 10.3390/pharmaceutics11110583

**Published:** 2019-11-07

**Authors:** Amanda Cherwin, Shelby Namen, Justyna Rapacz, Grace Kusik, Alexa Anderson, Yale Wang, Matey Kaltchev, Rebecca Schroeder, Kellen O’Connell, Sydney Stephens, Junhong Chen, Wujie Zhang

**Affiliations:** 1 BioMolecular Engineering Program, Physics and Chemistry Department, Milwaukee School of Engineering, Milwaukee, WI 53202, USA; cherwinae@msoe.edu (A.C.); namensl@msoe.edu (S.N.); rapaczj@msoe.edu (J.R.); kusikgo@msoe.edu (G.K.); andersonac@msoe.edu (A.A.); kaltchev@msoe.edu (M.K.); 13schrore@gmail.com (R.S.); kellen.d.oconnell@gmail.com (K.O.); sydneyjstephens@gmail.com (S.S.); 2 Mechanical Engineering Department, University of Wisconsin-Milwaukee, Milwaukee, WI 53211, USA; yalewang@uwm.edu (Y.W.); jhchen@uwm.edu (J.C.)

**Keywords:** artificial red blood cells, electrospinning and electrospray, pectin, oligochitosan, hydrogel, microcapsules

## Abstract

The goal of this research was to develop a novel oxygen therapeutic made from a pectin-based hydrogel microcapsule carrier mimicking red blood cells. The study focused on three main criteria for developing the oxygen therapeutic to mimic red blood cells: size (5–10 μm), morphology (biconcave shape), and functionality (encapsulation of oxygen carriers; e.g., hemoglobin (Hb)). The hydrogel carriers were generated via the electrospraying of the pectin-based solution into an oligochitosan crosslinking solution using an electrospinning setup. The pectin-based solution was investigated first to develop the simplest possible formulation for electrospray. Then, Design-Expert^®^ software was used to optimize the production process of the hydrogel microcapsules. The optimal parameters were obtained through the analysis of a total of 17 trials and the microcapsule with the desired morphology and size was successfully prepared under the optimized condition. Fourier transform infrared spectroscopy (FTIR) was used to analyze the chemistry of the microcapsules. Moreover, the encapsulation of Hb into the microcapsule did not adversely affect the microcapsule preparation process, and the encapsulation efficiency was high (99.99%). The produced hydrogel microcapsule system shows great promise for creating a novel oxygen therapeutic.

## 1. Introduction

In the United States, approximately 36,000 units of red blood cells (RBCs) are needed every day, according to American Red Cross. However, less than 38 percent of the population is eligible to give blood or platelets [1]. Donated blood undergoes costly screenings before it can be used to test for infectious diseases, such as HIV and hepatitis B and C [2]. Factors including eligible donors, costly testing, and limited shelf-life also impact the available supply. While blood donations will always be necessary, an oxygen therapeutic has the potential to help alleviate a number of the complexities associated with blood supply and demand. 

Different hemoglobin (Hb)-based oxygen therapeutics have been developed, but, unfortunately, no such product has been approved by the FDA for human use due to the toxicity of free hemoglobin, which can cause hypertension and cardiovascular dysfunction [3,4,5,6]. Nanoscale artificial oxygen transporter carriers, such as nanoparticles and liposomes, have been developed, showing promise in therapies such as wound healing and cancer treatment [7,8]. However, these carriers, even with PEGylation (which may lead to accelerated blood clearance (ABC)), tend to have a short circulation time compared with the 120 day circulation time of RBCs [6]. It is therefore critical to fully mimic the natural red blood cells, including their size and shape. Red-blood-cell-shaped carriers have been developed in recent years; for example, a polyelectrolyte microcapsule was produced using a red-blood-cell-shaped Ca(OH)_2_ template [9]. However, low encapsulation efficiency (oxygen transporter molecules are encapsulated inside the carrier, not attached to the carrier surface) remains an issue [5,9,10,11]. 

The purpose of this research is to develop a polymeric microcapsule system which mimics red blood cells to encapsulate oxygen transporters for use as an oxygen therapeutic. In particular, this study focuses on three criteria for the development of the microcapsule oxygen therapeutic: size, morphology, and functionality. In our previous studies [5,12], micro-scale red-blood-cell-shaped hydrogel capsules, using pectin and oligochitosan, were successfully developed and were shown to be able to encapsulate macromolecules. However, it is challenging to produce microcapsules/particles of less than 100 μm using traditional methods/equipment [13]. The PRINT^®^ technique has been used to fabricate red blood cell mimics but with a complex process [14]. The electrospinning setup for nanofiber production has been explored in order to generate microcapsules less than 10 μm in diameter through electrospray [15,16]. Electrospray offers such advantages as ease of upscaling and cost effectiveness. Very recently, electrospray based on an electrospinning setup has been successfully adopted to produce red-blood-cell-like microparticles [17,18]. As the electrospinning setup utilizes viscous liquids and a high voltage [19], pectin-based solution reformulation is necessary to increase the solution viscosity. The pectin-based solution and production process parameters were optimized through this research. Additionally, the impact of hemoglobin encapsulation on the key criteria and production process was explored. The result is a simplified and optimized production process of the pectin-based hydrogel microcapsules through formulation and parameter analysis. A pectin-based microcapsule encapsulating hemoglobin at the desired size and morphology was produced without adversely affecting the microcapsule preparation process. 

## 2. Materials and Methods 

### 2.1. Materials 

Low methoxy (LM) pectin (20.4% esterification) was purchased from WillPowder (Miami Beach, FL, USA). Pharmaceutical grade oligochitosan (95% deacetylation) of 2 kD molecular weight was obtained from Zhejiang Golden-Shell Pharmaceutical Co. Ltd. (Yuhuan, Zhejiang, China). All other chemicals were purchased from Sigma-Aldrich (St. Louis, MO, USA) and used without additional purification.

### 2.2. Preparation of Hydrogel Microcapsules

Hydrogel microspheres were prepared through electrospray by using an electrospinning setup (Linari Engineering, Valpiana, Italy). A 6–10% (*w/v*) pectin solution was sprayed into a 5% (*w/v*) oligochitosan solution (gelation solution) for approximately 10–15 min. The hydrogel microspheres were formed by the formation of pectin-oligochitosan electrolyte complexes. To obtain hemoglobin-loaded hydrogel microcapsules, hemoglobin powder was dissolved in a small volume of deionized (DI) water and then mixed with the pectin solution gently before electrospray [5]. The mixture was then sprayed into the oligochitosan solution to form loaded hydrogel capsules.

### 2.3. Optimization of Hydrogel Microcapsule Preparation Process 

Firstly, different concentrations of pectin were tested to determine the concentration to be used for the rest of the study. Moreover, preliminary testing was performed to select the parameters used for study as well as the working ranges for them (Table 1). Then, Design-Expert^®^ (Version 11; Stat-Ease Inc., Minneapolis, MN, USA) software was utilized to optimize the hydrogel microsphere preparation process. A Box–Behnken design (BBD) model was used. A total of 17 trials were run based on the design. Lastly, size and morphology were the responses for optimization. For the assessment of morphology, both size distribution and shape were considered and evaluated on a scale of 1–10. During optimization, the target size was between 5 and 10 μm with a morphology maximum rating of 10. 

### 2.4. Determination of Hemoglobin Encapsulation Efficiency

To determine the encapsulation efficiency, Hb-loaded microcapsules were prepared under the optimal condition. The encapsulation efficiency (EE) of hemoglobin within the capsules was determined by the difference between the initial amount of hemoglobin present and the unencapsulated hemoglobin in the supernatant:

(1) $$\mathrm{EE}=\frac{\mathrm{initial}-\mathrm{unencapsulated}}{initial}\times 100\%$$

A standard curve was generated, and hemoglobin concentrations were measured by using a UV–vis spectrophotometry (Evolution 60S; Thermo Fischer Scientific, Waltham, MA, USA) at 410 nm [5].

### 2.5. Characterization of the Hydrogel Microcapsules

Hydrogel microcapsules were dried in an oven and then Fourier transform infrared spectroscopy (FTIR; MIRacle 10, IR-Tracer 100; Shimadzu, Kyoto, Japan) was used to study the chemistry of the microcapsules.

## 3. Results and Discussion

### 3.1. Formulation of Hydrogel Microcapsules

In our previous studies [5,12], red-blood-cell-shaped microcapsules with diameters >300 μm, were successfully developed using a 3–4% (*w/v*) pectin solution through a vibration-based setup (minimum diameter of microcapsule/microbead which can be produced: 50 μm). Furthermore, a novel pectin-based nanofiber system was developed using an electrospinning setup. To reduce the microcapsule size, an electrospinning setup was chosen considering its capability to produce micro/nano-scale objects. Electrospinning, in general, involves a higher viscosity polymer solution and voltage compared with electrospray [19]. Both electrospray and electrospinning are based on similar principles. The major difference is the breaking of the jet, formed from the Taylor cone, into droplets during electrospray [19]. A pectin/PEO (viscosity enhancer)/glycerol (fluid modifier) mixture was tested and was able to produce red-blood-cell-shaped microcapsules less than 10 μm in size (data not shown). However, considering eventual industrial production and commercialization, a simple formulation is desired. To eliminate the viscosity enhancer and fluid modifier, 6–10% (*w/v*) pectin solutions were tested for their electrospray ability. When concentrations were lower than 7%, no biconcave-shaped microcapsules could be formed. On the other hand, the solution could not be sprayed when the concentration was higher than 9%. As a result, 8% was selected for formulating the hydrogel microcapsules.

### 3.2. Hydrogel Microcapsule Preparation Process Optimization

**Figure 1:**
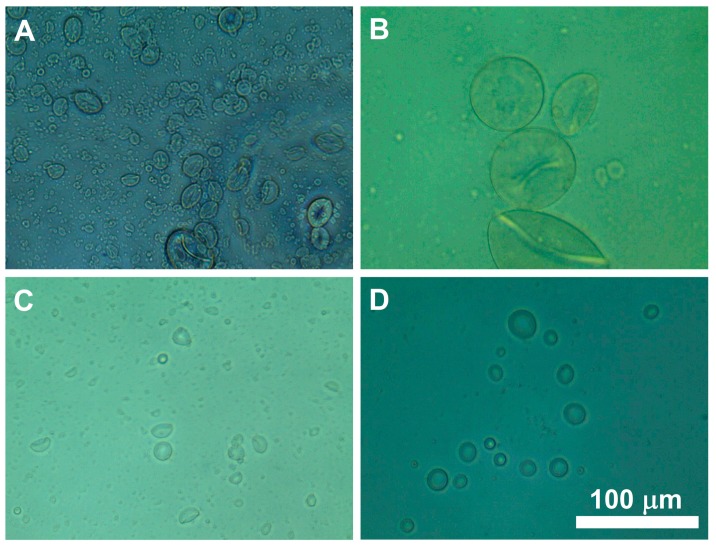
Representative images of microcapsules prepared during the optimization process. (**A**) Undesired shape and non-uniform size distribution; (**B**) Desired shape but large size; (**C**) Desired size but undesired shape; (**D**) Desired shape and size.

Based on the preliminary studies, voltage, flow rate, and height (from needle tip to the gelation solution (i.e., oligochitosan) surface) were found to have significant influences on the microcapsule preparation process and were chosen as the parameters for process optimization. As shown in Figure 1, the microcapsule morphology varied greatly when changing the process parameters. Table 1 describes the ranges of parameters explored to optimize the electrospray process and the Design-Expert^®^ software was used to apply a Box–Behnken model to outline the 17 trials to be tested. Quadratic models were utilized to represent the data with the complete quadratic model shown in Equation (2). The software was then used to analyze the resulting diameter and morphology of at least 200 microcapsules per trial. The desired responses were a diameter of less than 10 µm and a maximum morphology rating of 10. The quadratic model basis for the 17 trials from the Design-Expert^®^ software is shown below:

(2) $$Y=\beta_{0}\;+\;\sum_{i=1}^{k}\left(\beta_{i}X_{i}\right)\;+\;\sum_{i=1}^{k}(\beta_{i}X_{i}^{2})\;+\;\sum_{i=1}^{k-1}\sum_{j>i}^{k}\beta_{ij}X_{i}X_{j},$$

Y is the value of the response variable, β_0_ is the intercept coefficient, the first β_i_ items are the linear coefficients, the second β_i_ items are the quadratic coefficients, and β_ij_ items are the coefficients of the interaction terms.

During the optimization process, the software returned the following equations based on the trial data input:

(3) $$\begin{array}{ll} Y1=\;-73.8429 & +3.3299A+\;3.8211B\;+\;3.4461C-0.04478AB\;+0.0199AC\\ & -\;0.1418BC\;-\;0.1132A^{2}-\;0.0299B^{2}-\;0.0201C^{2} \end{array}$$

(4) $$\begin{array}{ll} Y2=19.2689 & +\;1.6188A-2.8900B-0.4850C\;-\;0.1125AB-0.0438AC\\ & \;+\;7.0613E-17\;BC+\;0.0516A^{2}\;+\;0.1120\;B^{2}\;+\;0.0580C^{2} \end{array}$$

where Y1 and Y2 are size and morphology respectively, A, B, and C are the independent variables: height (cm), voltage (kV), and flow rate setting. Both models (Equations (3) and (4)) fit the model well, as the lack of fit is not significant with a *p*-value of 0.9913 and 0.1961, respectively.

As shown in Figure 1, images of microcapsules produced during the optimization experimentation were taken using an optical microscope (EVOS XL; Thermo Fisher Scientific, Waltham, MA, USA).

**Figure 2:**
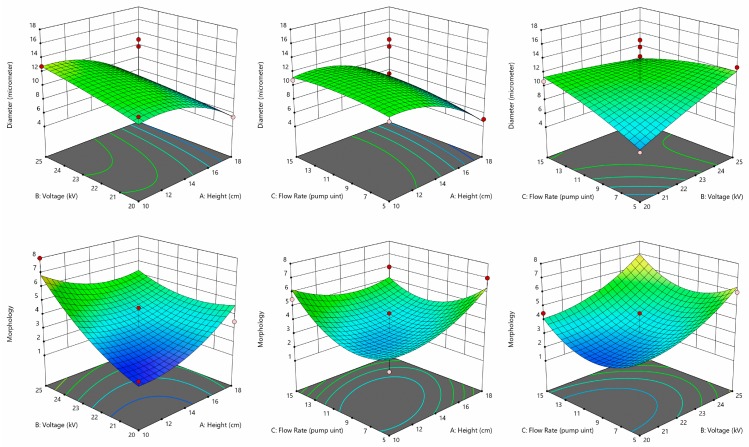
Response surface plots that show the effect of variables on the size (upper panel) and morphology (lower panel) before model reduction. The points which encompass the coordinates are displayed. Dark red dots: design points above predicted value; and pink dots: design points below predicted value.

Figure 2 shows the surface response curves generated by Design-Expert^®^ indicating the effects of two-factor interaction on capsule diameter and morphology. It was found that height (A) (*p* = 0.0328) produced a significant impact on microsphere size after model reduction by removing insignificant terms one at a time. The size of the droplet determines the size of the microcapsule. Under the same voltage, the electric field force decreases as the height increases [20,21]. However, the voltage had little effect on the microcapsule size, which might be due to the relatively narrow range of the voltage studied [22]. The significant terms found to impact the morphology are: B (*p* = 0.0095), AB (*p* = 0.0914) and C^2^ (*p* = 0.0361). These findings indicated that the morphology of microcapsules produced during electrospray shares a linear relationship with voltage and a quadratic relationship with the flow rate. During electrospray, droplet formation is driven mainly by the interplay between surface tension, gravity, and electric field force. As the voltage increases, the microcapsule morphology improves. This could be explained by the formation of a stable jet leading to monodisperse droplets when electrostatic force rather than gravitational force dominates the pulling force against surface tension [20]. At the same time, increasing the flow rate leads to a more stable jet but larger droplets [13,23]. Moreover, both height and voltage affect the electric field strength, which explains the significant influence of the interaction term AB on microcapsule morphology [21]. 

Considering both responses, to achieve the highest morphology rating as well as a diameter of 5–10 µm, the optimized parameters were determined to be a height of 13 cm, a voltage of 25 kV, and flow rate setting of 15 (2.1 mL/hr) as shown in Table 1. All further experimentation was conducted using the optimal parameters, thereby improving the process. 

### 3.3. Hemoglobin Encapsulation Efficiency

**Figure 3:**
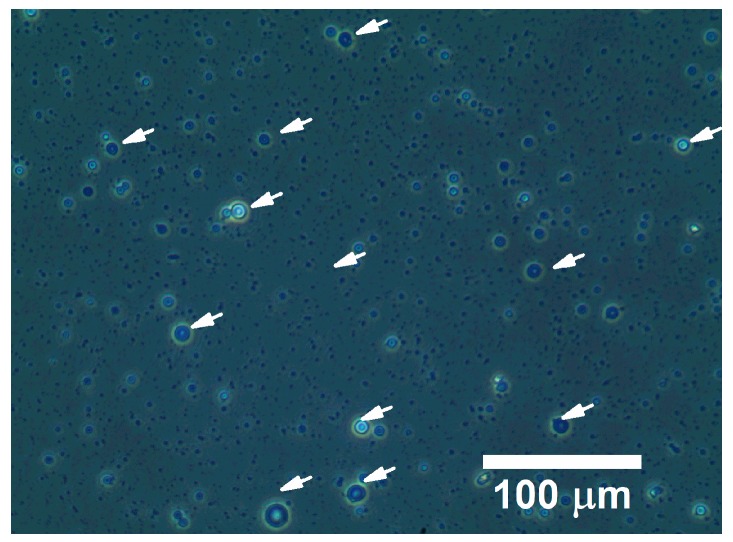
Image of hemoglobin (Hb)-loaded microcapsules (indicated by white arrows) prepared under the optimized condition.

The encapsulation of Hb within the microcapsules was also investigated. It was confirmed that the hemoglobin could be successfully encapsulated within these capsules through a passive loading process with a very high encapsulation efficiency of 99.99 ± 0.06%. Hb-loaded microcapsules (Microencapsulated Hb) are shown in Figure 3. It can be noticed that the microcapsules show a biconcave shape with uniform size distribution. The encapsulation did not negatively impact the hydrogel microcapsule formation. 

**Figure 4:**
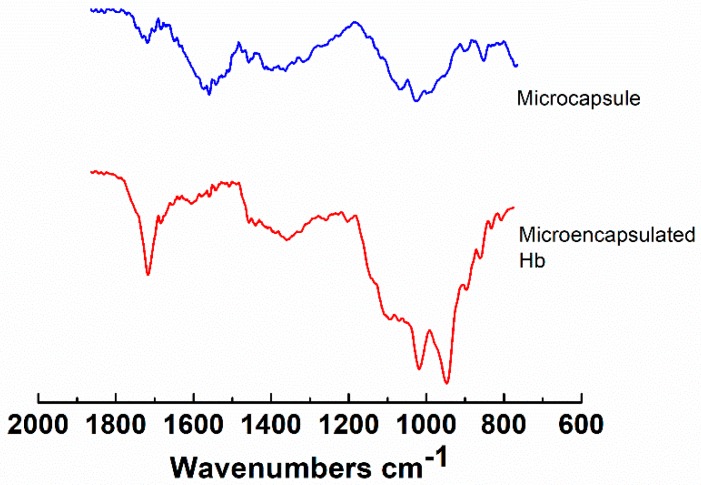
Fourier transform infrared (FTIR) spectra of hydrogel microcapsules and Hb-loaded microcapsules (microencapsulated Hb).

FTIR spectroscopy was also used to confirm the successful encapsulation of hemoglobin within the microcapsules. As shown in Figure 4, the presence of Hb is clearly evidenced by the significant changes that occur in the Amide I and Amide II regions around 1530–1650 cm^−1^. In particular, the peak around 1710 cm^−1^ becomes more pronounced due to the C=O stretching vibration of Hb [5,24]. This is also supported by the appearance of the strong peaks in the 1050–900 cm^−1^ region, which are typical for Hb [25]. 

## 4. Conclusions

An 8% pectin solution, without a viscosity-modifier, was chosen as the formulation for the hydrogel microcapsule production. This formulation enables a simple and quick preparation, which is essential during future industrial-scale production. Furthermore, the removal of the viscosity-modifying chemicals allows for concentration of the sample via centrifugation, which was previously impossible. The optimized condition was determined by using the Design-Expert^®^ software to produce microcapsules that are 5–10 µm in diameter and maintain a biconcave shape with a morphology resembling that of a natural red blood cell. Passive loading of hemoglobin into the microcapsules, confirmed by FTIR analysis, resulted in a high encapsulation efficiency of 99.99 ± 0.06%. 

Future work includes stability testing of the hydrogel microcapsule carrier as well as oxygen transport property. Alternatives to hemoglobin such as a synthetic gas carrier will also be explored.

## 5. Patent

Polymeric red-blood-cell-like particles (Inventors: Wujie Zhang, Rebecca Schroeder, Sydney Stephens, Haley Stephens, Kellen O’Connell, Nataline Duerig, Devon McCune, Jung Lee, and Gene A. Wright; Publication number: 20190183982; Publication date: 20 June, 2019; U.S. Patent).

## Figures and Tables

**Table 1:** Parameter optimization values. This table describes the optimized electrospray parameters determined using the Design-Expert^®^ software.

Parameter	Range	Optimized Value
Voltage (kV)	20–25	25
Flow Rate ^1^	5–15	15
Height (cm)	10–18	13

^1^ The units of the flow rates shown are specific to the pump used.
